# The possibility of chemical transformation of glucose in choline chloride/glucose deep eutectic solvent with thermal instability†

**DOI:** 10.1039/d4ra02546f

**Published:** 2024-05-28

**Authors:** Soki Idenaga, Takashi Hosoya, Hisashi Miyafuji

**Affiliations:** a Graduate School of Life and Environmental Sciences, Kyoto Prefectural University, Japan 1-5 Shimogamo-hangi-cho, Sakyo-ku Kyoto 606-8522 Japan miyafuji@kpu.ac.jp

## Abstract

Deep eutectic solvents (DESs), characterized by their low volatility, non-toxicity, and biodegradability, have gained attention as green solvents due to their minimal environmental impact and sustainability. The choline chloride/glucose DES, composed solely of biomass, is notable for its high biocompatibility and ability to be prepared at low cost. However, it is also known for its low thermal stability and tendency to denature when heated. In this study, we approached the choline chloride/glucose DES, with its thermal denaturation properties, as a unique chemical conversion medium entirely constituted from biomass. We investigated the thermal denaturation and reaction behaviors of the DES when subjected to prolonged heating. It was found that the choline chloride/glucose DES was relatively thermally stable at around 100 °C, but underwent thermal denaturation at 130 °C, enabling the production of 5-HMF and seven types of rare sugars derived from glucose. The yield of disaccharides containing seven types of rare sugars and 5-HMF relative to the weight of glucose was as high as approximately 70% and 5%, respectively. This study thus reveals that simply heating a liquid composed exclusively of biomass under mild conditions can generate a range of high-value compounds.

## Abstract 2

Choline chloride/glucose deep eutectic solvent is an environmentally friendly chemical reaction site that produces high value-added compounds.

## Graphical abstract

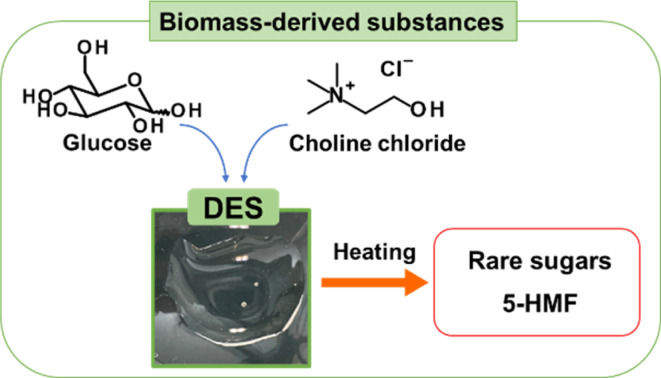

## Introduction

Deep eutectic solvents (DESs) are liquids comprised of a hydrogen bond donor (HBD) and a hydrogen bond acceptor (HBA). By heating the mixture of these two components to their eutectic point, DESs exhibit a significant reduction in melting point, allowing for the formation of DESs at temperatures substantially lower than the individual melting points of the HBD and HBA.^1^ DESs are distinguished by their low cost, high thermal stability, minimal volatility, and non-toxic nature. Given these characteristics, DESs have emerged as environmentally friendly solvents, drawing considerable interest for their reduced environmental impact in both preparation and application within chemical processes.^2^

The concept of DESs was initially introduced by Abbott *et al.* in 2003.^3^ In their seminal study, the choline chloride/urea DES was identified as demonstrating the most significant reduction in melting point, with the resultant DES melting at 12 °C. This is markedly lower than the melting (decomposition) points of its constituents: 302 °C for choline chloride, serving as the HBA, and 133 °C for urea, the HBD. Choline chloride (ChCl), in particular, was extensively examined for its capacity to lower melting points when combined with urea. Its effectiveness in this regard has led to its widespread adoption in subsequent research within this domain.^3,4^ As a component of DESs, ChCl—a vitamin B4 complex—also finds application as a nutritional supplement for livestock.^5^ It is lauded for its cost-effectiveness, non-toxicity, and high biocompatibility, making it an invaluable component of biomass-derived materials.^6,7^

As mentioned earlier, DESs, being easily adjustable due to their composition of two substances with hydrogen bonding capabilities, exhibit a variety of formulations. The revelation of their efficacy, particularly of those formulations incorporating metal salts in the extraction of metals^4,8^ has spurred extensive research into a diverse array of applications. These applications span from acting as CO_2_ capture solutions^9,10^ and electrolytes for lithium-ion batteries^11^ to facilitating the dissolution of pharmaceuticals,^12^ extraction of biomolecules^13,14^ desulfurization processes,^15^ and biomass processing.^16–19^ This breadth of utility underscores the versatility and potential of DESs in addressing a wide range of technological and environmental challenges.

Within the diverse spectrum of DESs, natural deep eutectic solvents (NADESs) stand out due to their high biocompatibility.^20^ These solvents are primarily composed of primary metabolites, including sugars, amino acids, organic acids, and choline derivatives. A notable example of NADESs is the ChCl/glucose DES, which is recognized for its high biocompatibility and cost-effective production.^21^ Glucose, a key component of this DES, is a monosaccharide known for its exceptional biocompatibility and presence in non-edible woody biomass, such as cellulose. In 2013, Hayyan *et al.* explored the preparation conditions and physical properties of ChCl/glucose DES.^22^ In 2018, N. Delgado-Mellado *et al.* prepared various ChCl-based DESs and reported on their thermal stability. In their report, it was stated that ChCl/glucose DES exhibited the highest thermal stability, showing only about 7 wt% mass loss after being heated at 403.2 K for 20 h.^23^ Further, in 2022, Marchel *et al.* investigated the thermal instability of DESs formulated with choline chloride. Their findings revealed discoloration of the ChCl/glucose DES at temperatures of 120 °C after 2 h of heating, with the formation of 5-hydroxymethylfurfural (5-HMF) being confirmed.^24^

The literature on the synthesis and thermal stability of ChCl/glucose DES remains limited. Within the scope of existing research, it is recognized that the thermal stability of this DES is influenced by the ratio of glucose to ChCl and the specific heating conditions applied. Yet, to date, no parameters have been established under which this DES maintains its integrity without undergoing thermal decomposition. This fact underscores a fundamental challenge: while ChCl/glucose DES can be synthesized, it is inherently susceptible to thermal-induced transformations, potentially yielding different chemical entities upon heating. Viewing ChCl/glucose DES from an alternative perspective, it represents an innovative, solvent-free chemical reaction medium, entirely derived from biomass materials. Notably, this DES exists in a liquid state, distinguishing it from simple solid mixtures and facilitating its function as a uniform reaction medium. Building on these considerations, the present study is designed to meticulously explore the thermal alteration and reaction dynamics of ChCl/glucose DES under extended heating conditions. This investigation is poised to illuminate its viability as a groundbreaking platform for chemical transformations within biomass, potentially heralding new avenues in green chemistry.

## Experimental

### Chemicals

ChCl and glucose were sourced from Wako Pure Chemical Industries, Ltd. The trimethylsilyl (TMS) reagent, *N*,*O*-bis(trimethylsilyl)trifluoroacetamide with 1% trimethylchlorosilane, was procured from Tokyo Chemical Industry Co., Ltd.

### Preparation of ChCl/glucose DES

ChCl and glucose, utilized in the heating experiments, were dried for 4 h at a pressure below 20 hPa while being heated separately at 50 °C. Subsequently, they were employed in the heating experiments. To prepare the ChCl/glucose DES, ChCl and glucose were mixed in various molar ratios as outlined in Table 1, achieving a total mass of 0.5 g. This mixture was transferred to a round-bottom flask, which was subsequently evacuated to 10–20 hPa using a diaphragm pump. The evacuated flask was placed in an oil bath preheated to a temperature range of 60–130 °C. Following a heating period of 30 min, the condition of the contents was visually examined to assess their transformation.

**Table 1:** The state of mixture of ChCl and glucose after heating at various temperatures^a^

Mixing ratio (mol/mol)		State							
ChCl/glucose		60 °C	70 °C	80 °C	90 °C	100 °C	110 °C	120 °C	130 °C
10/0	(=1/0)	S	S	S	S	S	S	S	S
9/1	(=1/0.1)	S	S	pL	pL	pL	pL	pL	S (brown)
8/2	(=1/0.25)	S	S	pL	pL	pL	pL	pL	S (brown)
7/3	(=1/0.43)	S	pL	pS	pS	pS	pS	L	L (brown)
6/4	(=1/0.67)	S	pL	pS	pS	pS	pS	L	L (brown)
5/5	(=1/1)	S	pL	pS	pS	pS	L	L	L (brown)
4/6	(=1/1.5)	S	pL	pS	pS	pS	L	L	L (brown)
3/7	(=1/2.3)	S	pL	pS	pS	pS	pS	L	L (brown)
2/8	(=1/4)	S	pL	pS	pS	pS	pS	pS	L (brown)
1/9	(=1/9)	S	pL	pL	pL	pL	pL	pL	L (brown)
0/10	(=0/1)	S	S	S	S	S	S	S	L (brown)

a S: solid, pL: partly liquid, pS: partly solid, L: liquid, S (brown): solid (brown), L (brown): liquid (brown).

### Thermal treatment of ChCl/glucose DES

ChCl (437 mg) and glucose (563 mg) – resulting in a mixture containing the two compounds at a 1/1 molar ratio – were introduced into a test tube and thoroughly mixed. The mixture was then placed in an oil bath preheated to 100–130 °C for a duration ranging from 0 to 180 min. Control experiments were also conducted with 563 mg of glucose in the absence of ChCl.

### Analyses of DES

Following the above thermal treatment, the obtained ChCl/glucose DES was analyzed using gel permeation chromatography (GPC) to elucidate its chemical composition. The preparation of samples for GPC analysis involved diluting the DES with 25 mL of ultrapure water and subsequently filtering the solution through a 0.45 μm filter. The filtrate was then subjected to GPC analysis using a Shimadzu Prominence system equipped with a Shodex SUGAR KS-802 column. The analysis was performed under the following conditions: the column temperature was maintained at 80 °C, and the system utilized both a refractive index detector (RID) and a photodiode array detector (PDA) for detection. Ultrapure water served as the eluent, with a flow rate set at 1.0 mL min^−1^.

For the determination of molecular weight (MW) distribution, a series of standard substances were employed, including maltose (MW: 342), isomaltotriose (MW: 504), isomaltotetraose (MW: 666), isomaltopentaose (MW: 828), and isomaltoheptaose (MW: 1150). The analysis aimed to quantify the glucose residual rate (%), oligomer yield (%), and 5-HMF yield (%) within the DES. The quantification was based on GPC chromatograms and calibration curves derived from the standard samples. Specifically, the production of oligomers was estimated by identifying the chromatogram peak near the MW of 342 as indicative of oligomers and applying a calibration curve constructed from the maltose standard.

1 Glucose residual rate (%) = (residual amount of glucose in any treatment time (g))/(initial amount of glucose (g)) × 100

2 Oligomer yield (%) = (amount of oligomers produced in any treatment time (g))/(initial amount of glucose (g)) × 100

3 5-HMF yield (%) = (amount of 5-HMF produced in any treatment time (g))/(initial amount of glucose (g)) × 100

To identify the compounds in the DES, gas chromatography-mass spectrometry (GC-MS) analysis was performed utilizing a Shimadzu GCMS-QP2010 Ultra gas chromatograph mass spectrometer. The preparation of samples for GC-MS analysis, a specific heating experiment was conducted. ChCl and glucose were mixed in a 1/1 molar ratio to yield a total mass of 1.0 g. This mixture was then heated at 130 °C for 180 min using an oil bath. This condition was selected because it exhibited the highest peak area ratio near the MW of 342, in comparison to the peak area of glucose. The thermally treated DES was dissolved in 1 mL of pyridine, followed by the addition of 0.3 mL of the TMS reagent. This mixture was then subjected to heating at 70 °C for 1 h to ensure complete derivatization. After heating, the sample was filtered through a 0.45 μm filter to remove any insoluble residues. The GC-MS analysis was conducted under following conditions. The analytical column used was a DB-5ms, characterized by a length of 30 m and an inner diameter of 0.25 mm. The injection port temperature was set at 250 °C. A temperature programming method was employed for the column oven, starting at 50 °C and then ramping to 320 °C, where it was held for 54 min, followed by a maintenance period at 320 °C for an additional 15 min. Helium was utilized as the carrier gas with a flow rate of 2.04 mL min^−1^. The emission current was set at 20 μA, with an ionization time of 2.0 ms.

During the preparation of samples for GPC, a dark-colored solid residue, insoluble in ultrapure water, was observed. This residue, henceforth referred to as the DES-water-insoluble portion, prompted further investigation to elucidate its chemical nature. To facilitate this analysis, a Hitachi TM3030PLUS Miniscope scanning electron microscope, equipped with a Quantax70 energy dispersive X-ray spectrometer (EDX), was employed. To obtain the DES-water-insoluble portion for EDX analysis, a specific heating experiment was conducted. ChCl and glucose were mixed in a 1/1 molar ratio to yield a total mass of 1.0 g. This mixture was then heated at 160 °C for 180 min using an oil bath. Following the heating process, the resultant DES was subjected to suction filtration through a membrane filter with a pore size of 0.5 μm, with ultrapure water used for washing. The filtered, insoluble portion was subsequently dried in a constant-temperature oven at 105 °C for 24 h to ensure complete dryness. The EDX analysis of the DES-water-insoluble portion was performed under conditions to achieve detailed elemental composition insights. An acceleration voltage of 5 eV and an analysis duration of 166 s were applied. The yield of the DES-water-insoluble portion was determined using the equation denoted as (4) in the study.

4 DES-water-insoluble portion yield (%) = (amount of DES-water-insoluble portion (g))/(initial amount of glucose (g))

## Results and discussion

### Preparation of ChCl/glucose DES

**Fig. 1:**
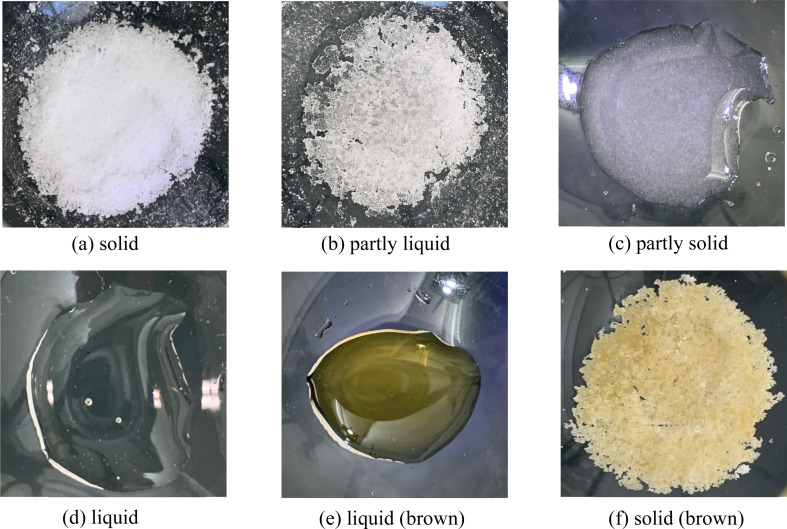
The pictures of ChCl and glucose mixture after heating at various temperature. (a) Solid: no change, where the mixture retains its white powdered form, (b) partly liquid: partial liquefaction, (c) partly solid: a liquid phase with residual solid particles, (d) liquid: a colorless transparent liquid, (e) liquid (brown): a brown transparent liquid, and (f) solid (brown): a brown solid.

ChCl and glucose are both colorless solids at room temperature. When these compounds were combined in their powdered forms under ambient conditions, the mixture predictably remained a colorless solid. Upon heating within a specified temperature range (80–130 °C), the ChCl and glucose mixture transitioned through six distinct states, contingent upon the molar ratio of the two compounds and the applied heating temperature. These states are depicted in Fig. 1 and encompass: (a) no change, where the mixture retains its white powdered form, (b) partial liquefaction, (c) a liquid phase with residual solid particles, (d) a colorless transparent liquid, (e) a brown transparent liquid, and (f) a brown solid. For clarity, these states have been classified as solid, partly liquid, partly solid, liquid, liquid (brown), and solid (brown), respectively.

Table 1 summarizes the physical states resulting from heating mixtures of ChCl and glucose at various molar ratios to different temperatures. Mixtures of ChCl and glucose in molar ratios of 5/5 (=1/1) and 4/6 (=1/1.5), upon heating to 110 °C, transformed into colorless, transparent liquids. The melting point of ChCl is 302 °C, while that of glucose is 145 °C. Hence, since the resulting liquid did not solidify upon cooling to room temperature, it is inferred that its melting point is below room temperature. This indicates that under these conditions, a ChCl/glucose DES is formed. Mixtures at molar ratios of 7/3 (=1/0.43), 6 : 4 (=1/0.67), and 3/7 (=1/2.3) also transitioned to DESs at 120 °C. However, upon increasing the temperature to 130 °C, these DESs transformed into brown transparent liquids, hinting at some form of alteration or decomposition within the DES at this elevated temperature. Thus, it was deduced that the formation of ChCl/glucose DES is contingent upon specific molar ratios and heating temperatures. Notably, at 130 °C, all DESs exhibited a color change to brown, suggesting a significant likelihood of chemical alteration or degradation. These findings underscore the thermal instability of ChCl/glucose DES. To further explore these thermal effects, subsequent experiments focused on the DES prepared from a 1/1 molar ratio of ChCl to glucose (5/5), examining the changes induced by heating.

### GPC analysis of the compounds solubilized in water

**Fig. 2:**
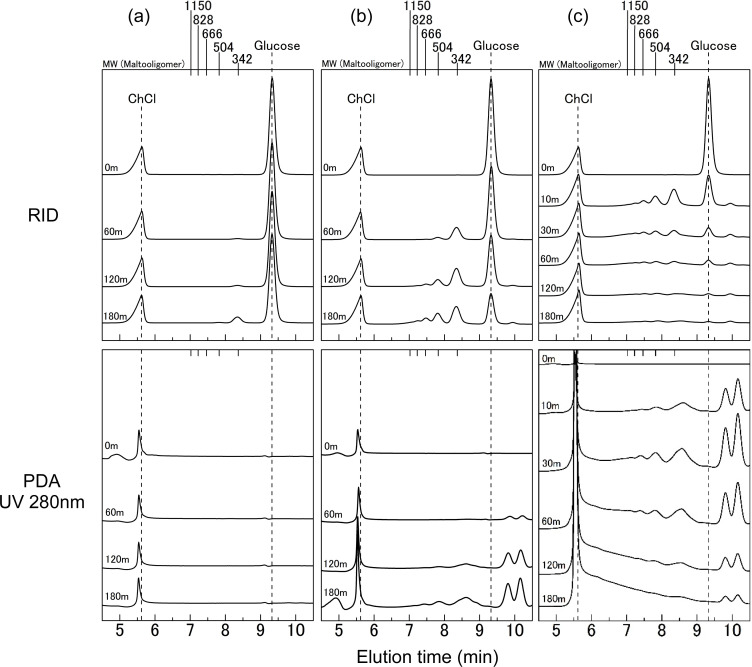
GPC chromatograms of ChCl/glucose DES after heating at 100 °C (a), 130 °C (b), 160 °C (c) for various treatment times. Top: RID. Bottom: PDA (UV 280 nm).

Fig. 2 illustrates the GPC analysis outcomes for the ChCl/glucose DES (1/1 molar ratio) subjected to heating for a specific duration. In the chromatograms obtained using the RI detector, as depicted in Fig. 2a, the peak appearing around 5.5 min is attributed to ChCl, while glucose is identified by a peak near the 9.4 min elution time. Notably, ChCl is a salt with a MW of 139. In this analysis, the column employed is designed for sugar separation. Consequently, the elution time and MW of ChCl do not correspond, resulting in it being detected as a void peak. These chromatograms reveal that after 180 min of heating at 100 °C, a new peak emerges around the MW of 342, albeit without significant alterations in the peaks corresponding to ChCl and glucose. Conversely, the PDA detector chromatogram does not confirm the presence of a peak corresponding to the MW of 342. This discrepancy suggests that the heating of ChCl/glucose DES results in the formation of higher MW compounds than ChCl and glucose, which do not exhibit UV absorption at 280 nm. The MW of 342 is indicative of disaccharides, known for their absence of the UV absorption. Hence, the heating of the DES at 100 °C for 180 min points to the potential generation of disaccharides. Note that given that ChCl lacks the UV-absorbing chemical structures, the peak observed around 5.5 min in the PDA chromatograms is likely attributable to some impurity.

Fig. 2b displays the GPC chromatograms of ChCl/glucose DES (1/1 molar ratio) following heating at 130 °C. Within the RID chromatograms, peaks corresponding to MWs of 342 and 504 were observed after 60 min of heating. By the 180 min mark, an additional peak at an MW of 666 became evident. There was no noticeable alteration in the peak attributed to ChCl, whereas the glucose peak exhibited a diminishing trend over time. In the PDA chromatograms, multiple peaks with MWs below 180 were discernible around an elution time of 9.4 min after 60 min of heating, and these peaks intensified with prolonged heating. Commencing from 120 min of heating, peaks corresponding to MWs of 342, 504, and 666 were also noted. These findings suggest that within the initial 60 min of heating, compounds that do not exhibit UV absorption at 280 nm and have MWs larger than those of ChCl and glucose are produced. Concurrently, glucose appears to decompose, yielding low MW compounds that exhibit the UV absorption. After 120 min of heating, the formation of compounds with UV absorption at 280 nm and larger MWs than ChCl and glucose is indicated.

As depicted in RID chromatograms in Fig. 2c, upon heating at 160 °C for a duration of 10 min, peaks were detected at MWs of 342, 504, 666, and higher than 666. Extending the heating time to 30 min resulted in the observation of peaks at MWs exceeding 1150. Notably, the intensity of the glucose peak diminished substantially over time, with peaks at MWs below 180 emerging as early as 10 min. After a heating duration of 120 min, an increase in the void peak was recorded. In the PDA chromatograms, peaks corresponding to MWs of 342, 504, 666, and higher than 666 were evident after only 10 min of heating. At 30 min, peaks at MWs above 1150 were observed, and a broad peak spanning the elution time between 6 and 9 min became apparent after 180 min of heating. These findings indicate that the reaction dynamics at 160 °C, as showcased in Fig. 2c, are markedly more pronounced than those observed in Fig. 2a and b. This suggests an accelerated rate of compound formation and decomposition at the higher heating temperature, resulting in a wider range of MWs and more significant changes in compound concentrations over time.

**Fig. 3:**
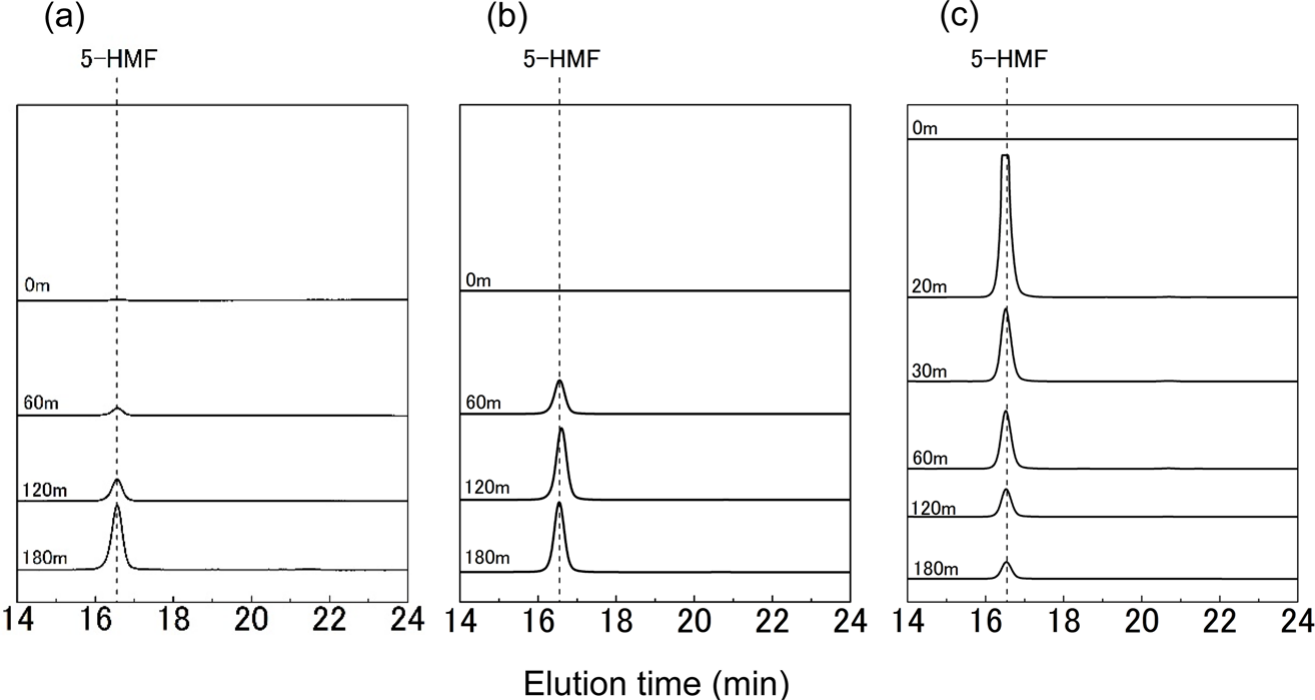
GPC chromatograms of ChCl/glucose DES after heating at 100 °C (a), 130 °C (b), 160 °C (c) for various treatment times. Detector: PDA UV 280 nm.

Fig. 3 presents GPC chromatograms with an emphasis on the elution time range of 14 to 24 min. A distinct peak attributed to 5-HMF was identified near an elution time of 16.5 min. It is known that 5-HMF is produced through the dehydration reaction of glucose.^25–27^ In this DES, it is suggested that dehydration decomposition of glucose is occurring. In Fig. 3a and b, there was a noticeable increase in the 5-HMF peak over time, indicative of an escalation in 5-HMF production. Conversely, Fig. 3c reveals a decline in the 5-HMF peak following 30 min of heating, suggesting a probable decrease in 5-HMF amount due to its further decomposition at an elevated temperature.

We also conducted kinetic analysis of glucose decomposition and 5-HMF formation. Our findings revealed that the glucose decomposition cannot be approximated by a pseudo-first-order model especially at 160 °C. The analysis further indicated that 5-HMF formation is only one of several possible pathways for glucose decomposition (refer to Fig. S1 in the ESI† and the accompanying discussion for details).

### Identification of compounds solubilized in water

**Fig. 4:**
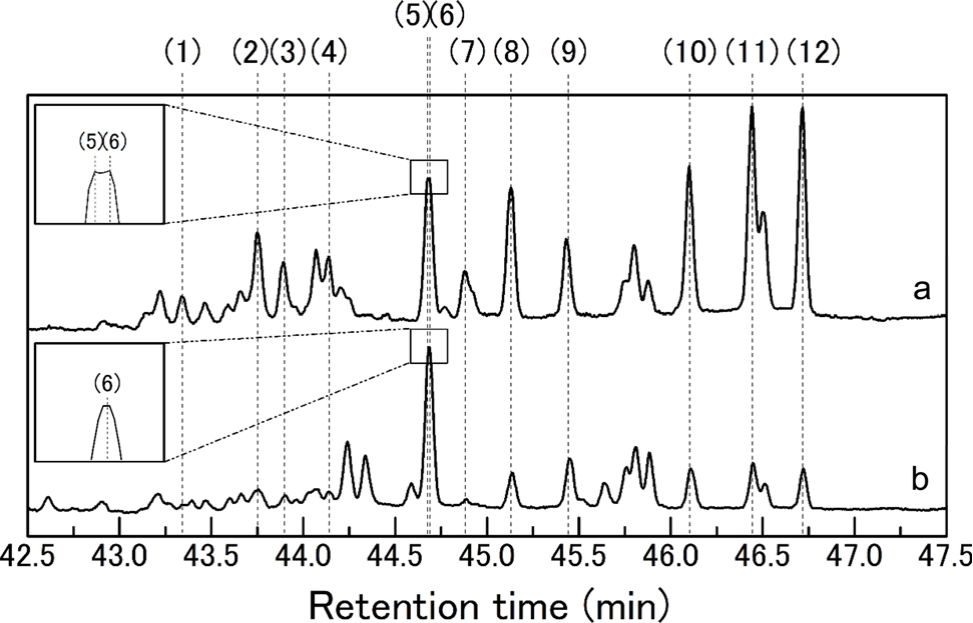
Total ion chromatograms obtained in the GC-MS analysis of (a) the trimethylsilylated ChCl/glucose DES and (b) the trimethylsilylated glucose after heating at 130 °C for 180 min.

To further elucidate the detailed reaction behaviors of the ChCl/glucose DES under thermal conditions, GC-MS analysis was conducted on samples subjected to heating. Fig. 4a illustrates the total ion chromatogram obtained from GC-MS analysis of the ChCl/glucose DES (1/1 molar ratio) heated at 130 °C for 180 min. Assigned numbers on the peaks correspond to compounds identified, with the specifics detailed in Table 2. Notably, peaks (5) and (6) are closely aligned in retention times, with an enlarged depiction provided for clarity. Table 2 lists the identified compounds, their retention times (RTs), and characteristic fragment ions, where all peaks numbered (1) to (12) were classified as disaccharides composed of two glucose units. It is known that disaccharides are formed through the dehydrative polymerization of glucose,^28^ and under the employed conditions, it is indicated that this type of polymerization of glucose is occurring. The disaccharides identified, including cellobiose, nigeroses, maltose, neotrehalose, laminaribiose, cellobiose (repeated for emphasis), kojibiose, sophorose, isomaltose, and gentiobiose, are illustrated in Fig. S1† with their chemical structures. These disaccharides are differentiated by the bonding positions between glucose units and the variations in α- and β-linkage patterns. As detailed in Table 2, certain disaccharides like cellobiose, nigeroses, and isomaltose are represented by two peaks each, reflecting the *α* and *β* configurations at their reducing terminal hydroxy groups. Some disaccharides that did not show two peaks reflecting the *α* and *β* configurations may exist in only one of the *α* or *β* configurations depending on the preparation of the standard used to identify the disaccharide or the preparation of this sample. In Fig. 4, peaks without numbers remain unidentified, yet the retention time span from 44.2 to 47 min showcases fragment ions similar to those of the identified disaccharides (see Fig. S2–S4 in ESI for details†). This observation strongly suggests that these unidentified peaks likely represent disaccharides not depicted in Fig. S1,† indicating a broad spectrum of disaccharide formation under the given thermal conditions.

**Table 2:** The list of identified disaccharides, their retention times (RTs), and their characteristic ions obtained in the GC-MS analysis of trimethylsilylated ChCl/glucose DES after heating at 130 °C for 180 min

No.	Disaccharides	RT (min)	Characteristic ions (*m*/*z*)
(1)	Cellobiose	43.34	73, 191, 204, 361
(2)	Nigerose	43.75	73, 129, 147, 204, 217, 361
(3)	Maltose	43.89	73, 191, 204, 217, 361
(4)	Nigerose	44.14	73, 129, 204, 217, 361
(5)	Neotrehalose	44.67	73, 103, 147, 191, 204, 217, 361
(6)	Laminaribiose	44.68	73, 129, 147, 204, 217
(7)	Cellobiose	44.88	73, 191, 204, 217, 361
(8)	Kojibiose	45.13	73, 147, 191, 204, 217, 361
(9)	Sophorose	45.43	73, 147, 191, 204, 217, 361
(10)	Isomaltose	46.10	73, 191, 204, 217, 361
(11)	Gentiobiose	46.44	73, 191, 204
(12)	Isomaltose	46.72	73, 191, 204, 361

The MW of disaccharides composed of glucose is consistently 342. Thus, the peaks observed around the MW of 342 at 180 min in the GPC chromatograms with the RI detector (Fig. 2b) are presumed to originate from these disaccharides. Notably, these peaks around the MW of 342 also exhibited UV absorption at 280 nm (Fig. 2b, PDA at 180 min). Given that the disaccharides illustrated in Fig. S1† lack the UV absorption, it is inferred that structures distinct from these disaccharides, potentially oligomers with the UV-absorbing features, are being formed.

In the 60 min treatment at 130 °C, as depicted in the RID chromatograms of Fig. 2b, peaks near the MW of 504 were observed, yet no peaks appeared in the PDA chromatogram of Fig. 2b. The MW of 504 corresponds to trisaccharides composed of glucose. Similarly, in the treatment at 100 °C shown in the RID chromatograms of Fig. 2a, the peak near an MW of 342 was noted, while this peak was absent in the PDA analysis. These findings indicate that, upon heating the ChCl/glucose DES, an initial-stage reaction takes place in which glucose undergoes dehydration polymerization, consequently resulting in the formation of oligosaccharides. As the reaction advances, various glucose-derived dehydration decomposition products, such as 5-HMF, are also produced. 5-HMF is known for its thermal instability and characteristic UV absorption, with 9% of its content being transformed when heated at 100 °C under acidic conditions.^29^ It is well-documented that 5-HMF can decompose into low molecular weight compounds, such as levulinic acid,^29^ or undergo polymerization to form polymers, including like humins.^30,31^ Consequently, during the thermal reaction of the ChCl/glucose DES, it can be inferred that as the reaction advances, a range of glucose-derived decomposition products, notably 5-HMF, initiate various reactions. These reactions lead to the formation of oligomers with higher MWs than glucose, which also exhibit UV absorption.

To further understand the characteristics of disaccharides formed by heating ChCl/glucose DES, glucose alone was heated at 130 °C for 180 min without ChCl, and GC-MS analysis was conducted similarly, with the total ion chromatogram shown in Fig. 4b. Peaks numbered correspond to disaccharides identified in Fig. 4b and Table 2. A comparison between the disaccharides produced by heating ChCl/glucose DES and those generated from heating glucose alone reveals that cellobiose and laminaribiose are unique to the ChCl/glucose DES treatment. This suggests ChCl's role in glucose polymerization, leading to the formation of specific disaccharides unattainable by heating alone. Thus, choline chloride/glucose DES represents a novel chemical transformation medium for deriving specific oligosaccharides from glucose, also acting as a parent solvent for these disaccharides due to their solubility.

### Quantification of glucose-derived products

**Fig. 5:**
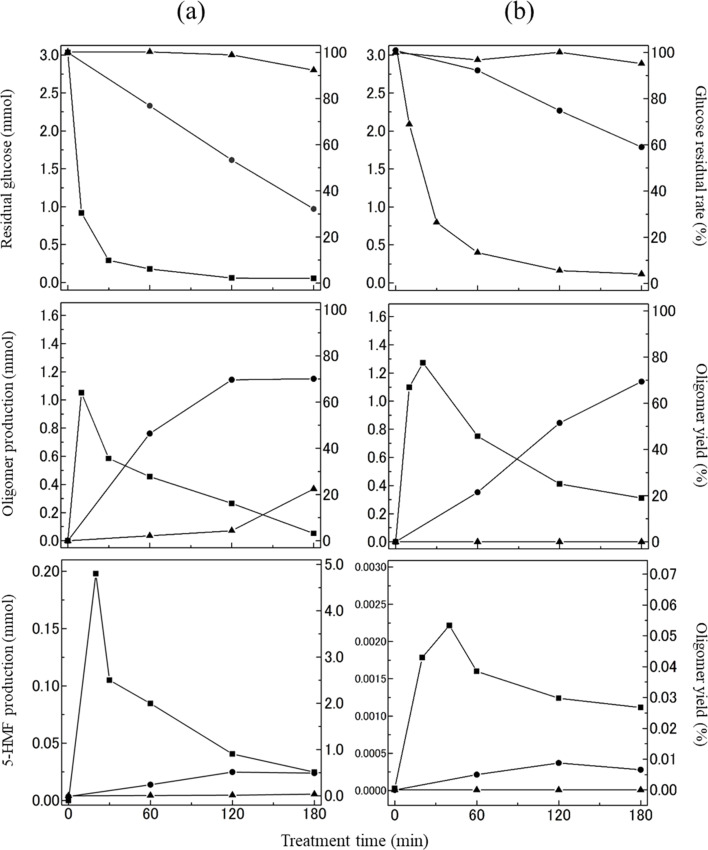
Residual rate of glucose and the yields of oligomer and 5-HMF, and the amount of each substance during the heating of ChCl/glucose DES (a) and neat glucose (b) at 100 °C (▲), 130 °C (●), and 160 °C (■). The left axis shows the number of moles of each substance, and the right axis shows the yield.

Based on the GPC chromatograms in Fig. 2 and 3, the glucose residual rate and the yields of the oligomers and 5-HMF, and the number of moles of each substance was conducted during the heating of the ChCl/glucose DES, revealing their time-dependent concentration changes as depicted in Fig. 5a. The detailed methodology for this quantification can be found in the experimental section. The specific recovery and yield values are summarized in Tables S1 and S2 of ESI.† At 100 °C, the glucose residual rate remained largely unchanged for up to 60 min. A minor decrease was observed at 120 min, and by 180 min, the rate had diminished to approximately 90% (2.80 mmol). The initial 60 min of heating exhibited minimal formation of oligomers and 5-HMF, suggesting a degree of thermal stability for the ChCl/glucose DES at this temperature. However, after 120 min, the formation of approximately 5% (0.37 mmol) oligomers indicated an advancing degradation of glucose due to prolonged heating. The decomposition of glucose became more rapid at 130 °C, with the glucose residual rate dropping to around 30% (0.97 mmol) after 180 min of heating. Concurrently, the yields of oligomers and 5-HMF progressively increased, reaching roughly 70% (0.40 mmol) and 0.5% (0.02 mmol), respectively, after 180 min. At a higher temperature of 160 °C, the glucose residual rate diminished more swiftly, with glucose nearly completely degraded after 120 min. The oligomer yield initially surged to about 65% (1.05 mmol) within the first 15 min but then declined, nearly vanishing after 180 min. The 5-HMF yield mirrored this pattern, peaking at approximately 4.3% (0.20 mmol) after 15 min before diminishing. This suggests that both oligomers and 5-HMF are unstable under these conditions, likely undergoing significant degradation or further reaction pathways.

Fig. 5b illustrates the temporal changes in the glucose residual rate and the yields of the oligomers and 5-HMF, and the number of moles of each substance when heating glucose without the addition of ChCl. Across all heating temperatures, the patterns in glucose residual rate and the yields of the oligomers and 5-HMF paralleled those depicted in Fig. 5a. A closer examination between Fig. 5a and b, particularly at 130 °C, reveals a lower residual rate of glucose when heated in the presence of ChCl, suggesting a more accelerated degradation of glucose within the ChCl/glucose DES than when glucose is heated independently. In terms of oligomer yields at 100 °C and 130 °C, an enhanced rate of increase was noticeable when heating the ChCl/glucose DES, in contrast to solely heating glucose. Additionally, the yield of 5-HMF in the ChCl/glucose DES demonstrated a more rapid increase and reached higher peaks compared to the outcomes of heating glucose alone. These observations underscore the influential role of ChCl in facilitating not only the accelerated degradation of glucose but also in promoting the formation and higher yields of oligomers and 5-HMF within the DES system. Both oligomers and 5-HMF are produced through the dehydration of glucose. Hayyan *et al.* reported that the pH of ChCl/glucose DES (molar ratio 1.5/1) decreased with increasing temperature. In their report, the pH of ChCl/glucose DES (molar ratio 1.5/1) is approximately 7 at room temperature. However, at 358.15 K, it decreases to around 6.^22^ Given the temperature conditions in this study, the ChCl/glucose DES becomes more acidic, potentially facilitating the formation of oligomers and 5-HMF through glucose dehydration.

The above finding revealed that ChCl/glucose DES demonstrated a degree of stability up to a certain point at 100 °C. However, upon elevating the temperature to 130 °C and 160 °C, significant dehydration polymerization and decomposition of glucose occurs, indicating accelerated deterioration. It became therefore apparent that ChCl/glucose DES demonstrated a higher susceptibility to thermal denaturation compared to glucose heated in isolation. This observation is underscored by the markedly higher yield of 5-HMF in the DES upon heating, which is approximately 100 times greater than the yield from heating glucose alone. Based on these observations, it is posited that ChCl acts catalytically in facilitating glucose's degradation through processes such as polymerization and decomposition.

### EDX analysis on DES-water insoluble portion

**Fig. 6:**
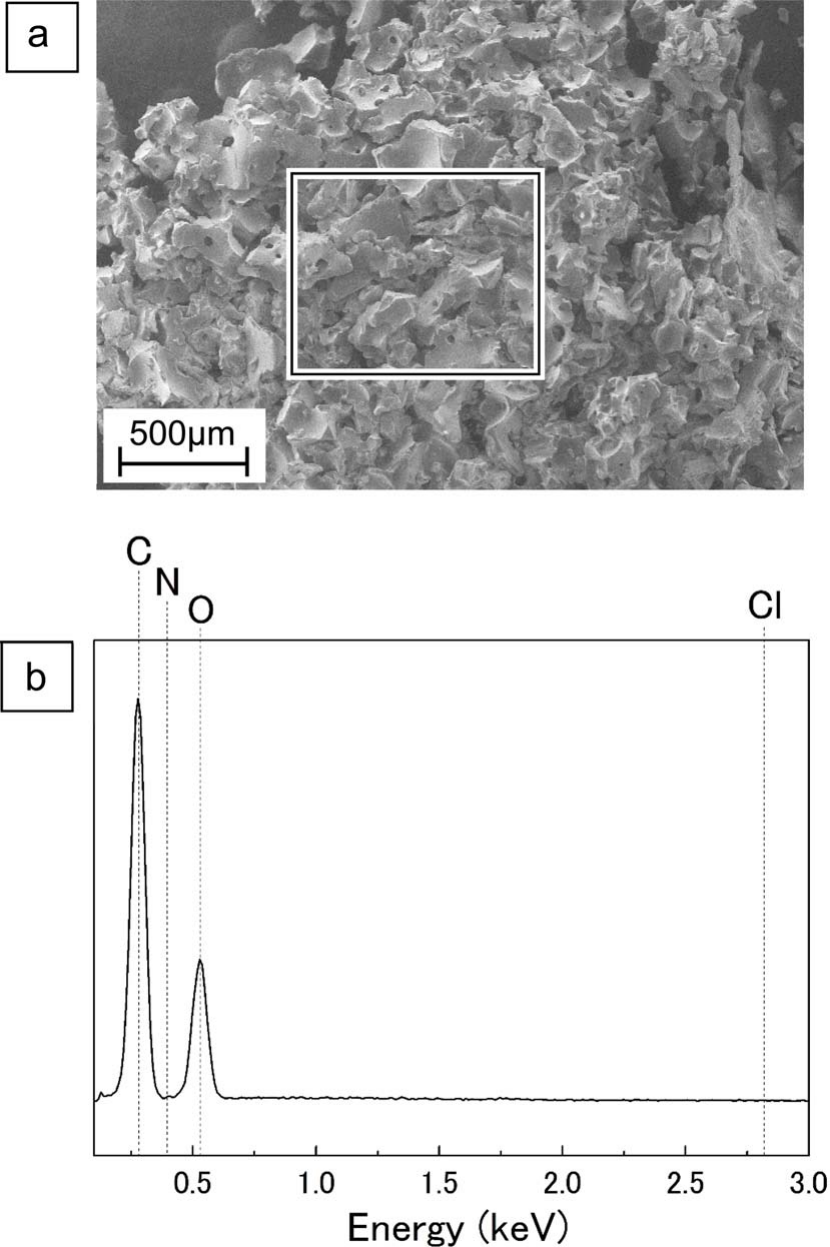
SEM-EDX analysis on DES-water insoluble portion after heating of ChCl/glucose DES at 160 °C for 180 min. (a) the SEM image. (b) The EDX spectrum on square area in (a).

Following the heating of the ChCl/glucose DES at 160 °C, the introduction of water led to the creation of a black DES-water-insoluble portion, as shown in Fig. S5 of ESI.† This insoluble portion was not observed at lower temperatures of 100 °C or 130 °C, indicating its unique formation at 160 °C. The yield of this DES-water-insoluble portion, detailed in Table 3, was observed to increase with time. A SEM image of the DES-water-insoluble portion are provided in Fig. 6a, with a specific area highlighted and analyzed using EDX, the result of which is depicted in Fig. 6b. The analysis revealed the presence of carbon (C) and oxygen (O) in the insoluble portion, with the notable absence of nitrogen (N) and chlorine (Cl). This absence implies that ChCl does not contribute to the composition of the DES-water-insoluble portion, leading to the inference that this fraction is primarily composed of compounds derived from glucose.

**Table 3:** The yield of water-insoluble portion after heating of ChCl/glucose DES at 160 °C for various treatment times

Treatment time (min)	Yield (wt%)
0	0
10	0.05
20	0.38
30	1.46
60	9.90
120	18.7
180	27.5

Examining GPC chromatograms heated at 160 °C, as shown in the PDA chromatograms of Fig. 2c, peak exceeding the exclusion limit beyond the elution time of near 5.5 min was observed. This result suggests the formation of high MW polymers with UV absorption at 280 nm. At 100 °C, as shown in Fig. 2a, no significant changes were observed in the peak near elution time 5.5 min, even with prolonged heating. At 130 °C, as shown in Fig. 2b, there was a slight increase, but no prominent peak similar to that observed at 160 °C was observed. Sugars such as glucose undergo complex chemical reactions such as decomposition and polymerization under acidic conditions upon heating, leading to the formation of humins, water-insoluble polymeric compounds with the UV absorption.^27,28^ As reported by Hayyan *et al.*, the pH of ChCl/glucose DES (molar ratio 1.5/1) decreased with increasing temperature.^22^ Therefore, ChCl/glucose DES during heating is considered acidic, and at 160 °C, glucose in the DES is presumed to undergo complex chemical reactions such as decomposition and polymerization, resulting in the generation of humins. Although the generated humins were initially soluble in the DES, they appeared to precipitate upon the addition of water. Due to the minimal formation of humins during heating at 100 °C and 130 °C, it is inferred that no precipitation occurred.

## Conclusions

The preparation of ChCl/glucose DES was successfully achieved using specific molar ratios and heating at approximately 100 °C. This DES exhibits thermal denaturation characteristics, as heating facilitated the dehydration polymerization and decomposition of glucose, leading to the formation of 5-HMF and various oligomers, including disaccharides. Prolonged heating further resulted in the production of humins. Among the disaccharides formed, rare sugars such as isomaltose, nigerose, kojibiose, neotrehalose, laminaribiose, gentiobiose, and cellobiose were identified. These findings highlight that while ChCl/glucose DES may not exhibit high thermal stability, it acts as an effective reaction medium for generating a diverse range of valuable oligosaccharides.

## Conflicts of interest

There are no conflicts to declare.

## Supplementary Material

RA-014-D4RA02546F-s001
