# Supplementary material for: The possibility of chemical transformation of glucose in choline chloride/glucose deep eutectic solvent with thermal instability
Source: RSC Adv. 2024 May 28;14(24):17022–31. doi: 10.1039/d4ra02546f (PMC11130762; doi:10.1039/d4ra02546f)
Supplement: RA-014-D4RA02546F-s001 [file RA-014-D4RA02546F-s001.pdf]

Electronic Supplementary Material (ESI) for RSC Advances.
This journal is © The Royal Society of Chemistry 2024

Supporting information

The possibility of chemical transformation of glucose in choline chloride/glucose deep eutectic solvent

Soki Idenaga, Takashi Hosoya, Hisashi Miyafuji*

Graduate School of Life and Environmental Sciences, Kyoto Prefectural University, Japan. 1- 5 Shimogamo-hangi-cho, Sakyo-ku, Kyoto 606-8522, Japan

*Corresponding author: Email address: miyafuji@kpu.ac.jp

Contents

Kinetic curves of glucose and 5-HMF (Fig. S1) S2

The structure of identified disaccharides obtained in the GC-MS analysis (Fig. S2) S3

The mass spectra of identified disaccharides obtained in the GC-MS analysis of the trimethylsilylated ChCl/glucose DES (Figs. S3 and S4) S4-5

The mass spectra of unidentified peaks observed in the GC-MS analysis of trimethylsilylated ChCl/glucose DES (Fig. S5) S6

Glucose residual rate (%) and the yield (wt%) of major glucose-derived products obtained upon heating neat glucose ChCl/glucose DES (Tables S1) and neat glucose (Tables S2) S7

The water-insoluble portion after heating of ChCl/glucose DES (Fig. S6) S8

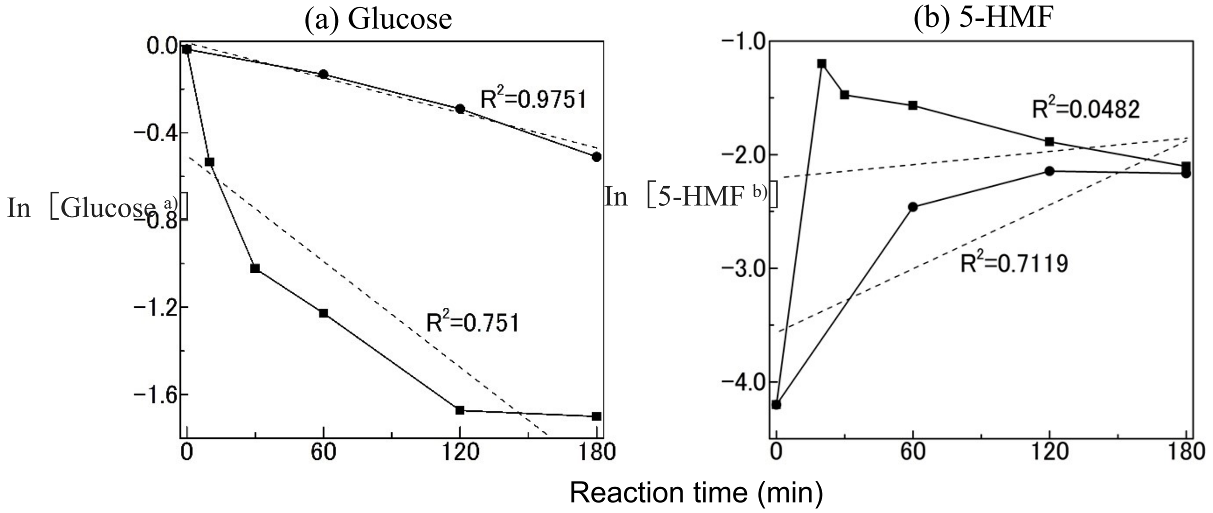

Fig. S1. Kinetic curves of glucose (a) and 5-HMF (b) at 130℃ (●), 160℃ (■).

a) Glucose = glucose (mmol)/ChCl (mmol), b) 5-HMF = 5-HMF (mmol)/ChCl (mmol)

As shown in Fig. S1, the kinetic curves (In [Glucose], In [5-HMF]) were derived from the GPC analysis results, and a pseudo-first-order analysis was performed. In this analysis, we defined the concentrations of glucose and 5-HMF as their relative molar amount to that of ChCl (3.13 mmol), which is assumed to remain constant during heat treatment. Pseudo-first-order analysis was not conducted for the sample heated to 100°C, as there was negligible glucose decomposition and 5-HMF production.

As illustrated in Fig. S1a, the kinetic curve for glucose decomposition seemed to be approximated by a pseudo-first-order model at 130°C, as suggested from a liner relationship between ln[Glucose] and reaction time. However, it becomes obvious that this approximation broke down at 160°C. As discussed in the text, the GPC chromatograms in Fig. 2 suggested that the multiple peaks observed, apart from those of choline chloride and glucose, were likely due to compounds generated from glucose decomposition, indicating that glucose decomposition fundamentally involves many reaction pathways including the formation of the oligomer (mainly disaccharides), 5-HMF, etc. The above kinetic analysis may suggest that glucose decomposition is explainable roughly by combination of several pathways that can be approximate by the pseudo-first-order model. In contrast, at 160°C, glucose decomposition seems to involve pathways that cannot be approximated by the pseudo-first-order model. This failure of such approximation might be explained by the idea that polymerization pathway presumed to be second order becomes dominant at elevated temperatures.

Additionally, the kinetic curves of 5-HMF at 130°C and 160°C did not conform to a simple semi-logarithmic plot. This outcome is typical when a reactant (glucose) decomposes via multiple pathways that compete with the 5-HMF formation, suggesting that 5-HMF formation is only one of several glucose decomposition pathways. This finding supports our statement that the multiple peaks observed in the GPC chromatogram are likely due to compounds produced by glucose decomposition via various pathways.

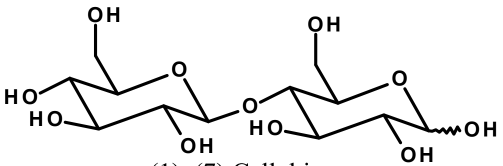

(1), (7) Cellobiose

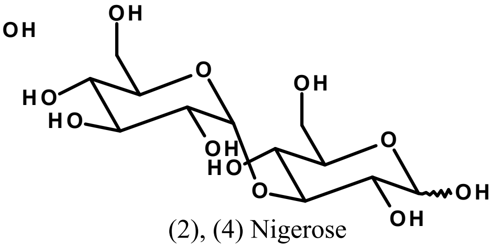

(2), (4) Nigerose

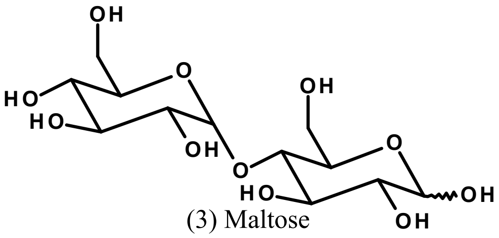

(3) Maltose

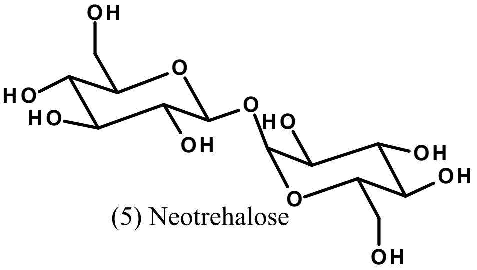

(5) Neotrehalose

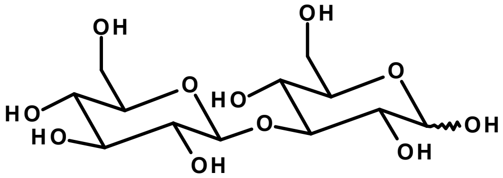

(6) Laminaribiose

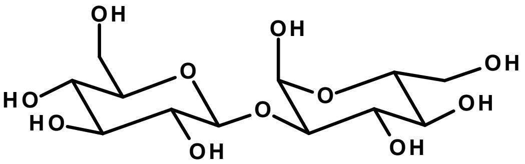

(9) Sophorose

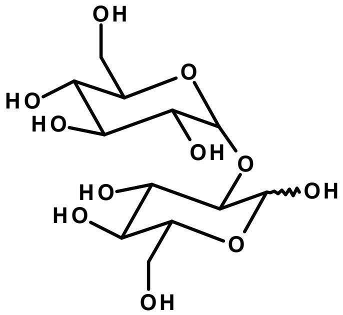

(8) Kojibiose

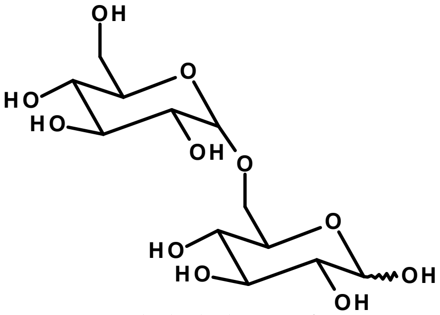

(10), (12) Isomaltose

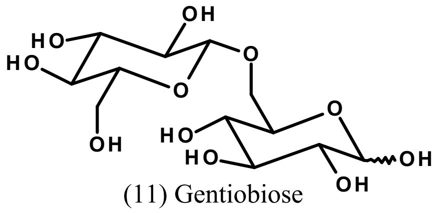

(11) Gentiobiose

Fig. S2. The structure of the identified disaccharides obtained in the GC-MS analysis of trimethylsilylated ChCl/glucose DES (1:1 molar ratio) after heating at 130°C for 180 min.

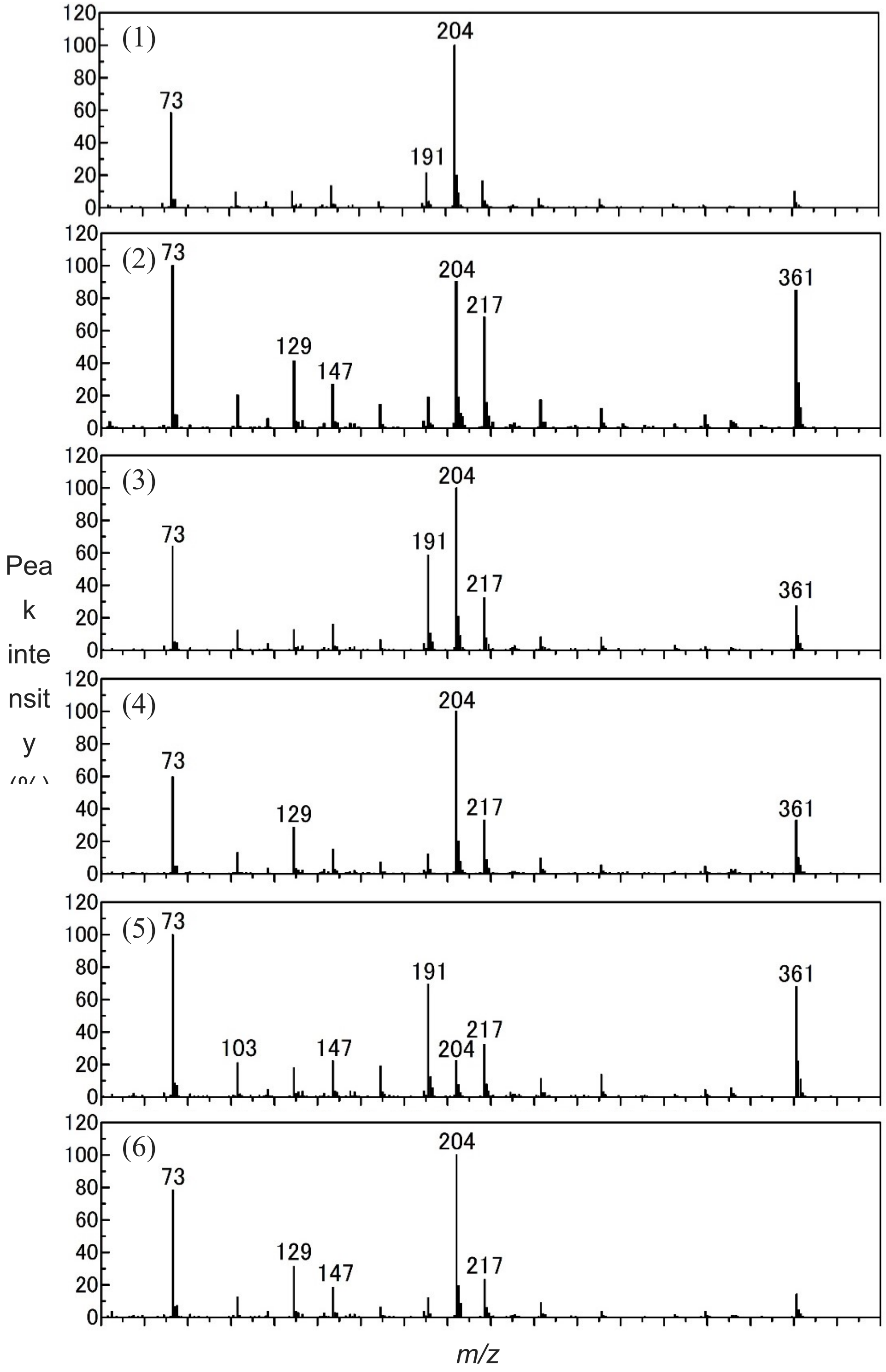

Fig. S3. The mass spectra of the identified disaccharides (1) -(6) obtained in the GC-MS analysis of trimethylsilylated ChCl/glucose DES (1:1 molar ratio) after heating at 130°C for 180 min.

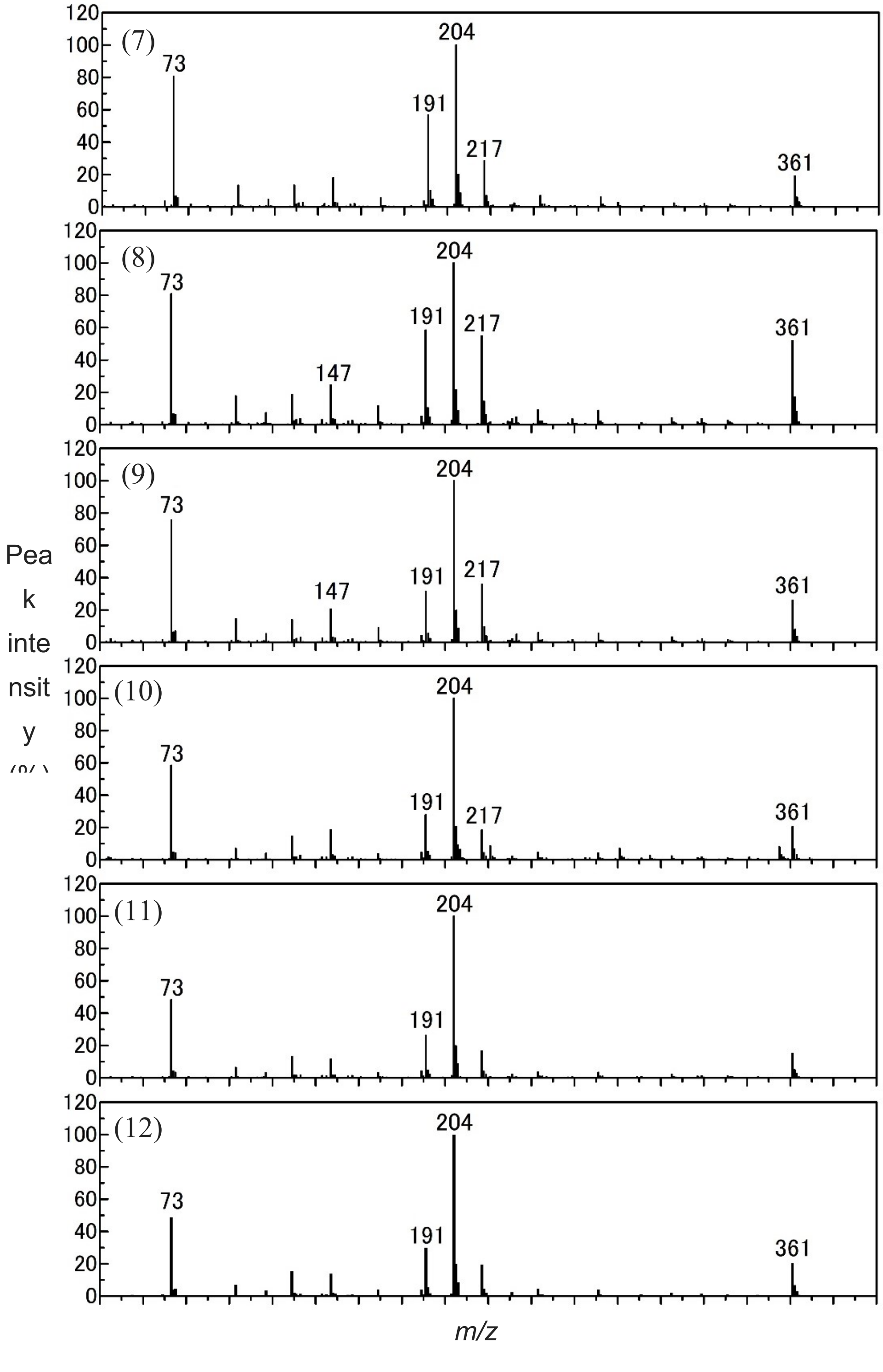

Fig. S4. The mass spectra of identified disaccharides (7) -(12) obtained in the GC-MS analysis of trimethylsilylated ChCl/glucose DES (1:1 molar ratio) after heating at 130°C for 180 min.

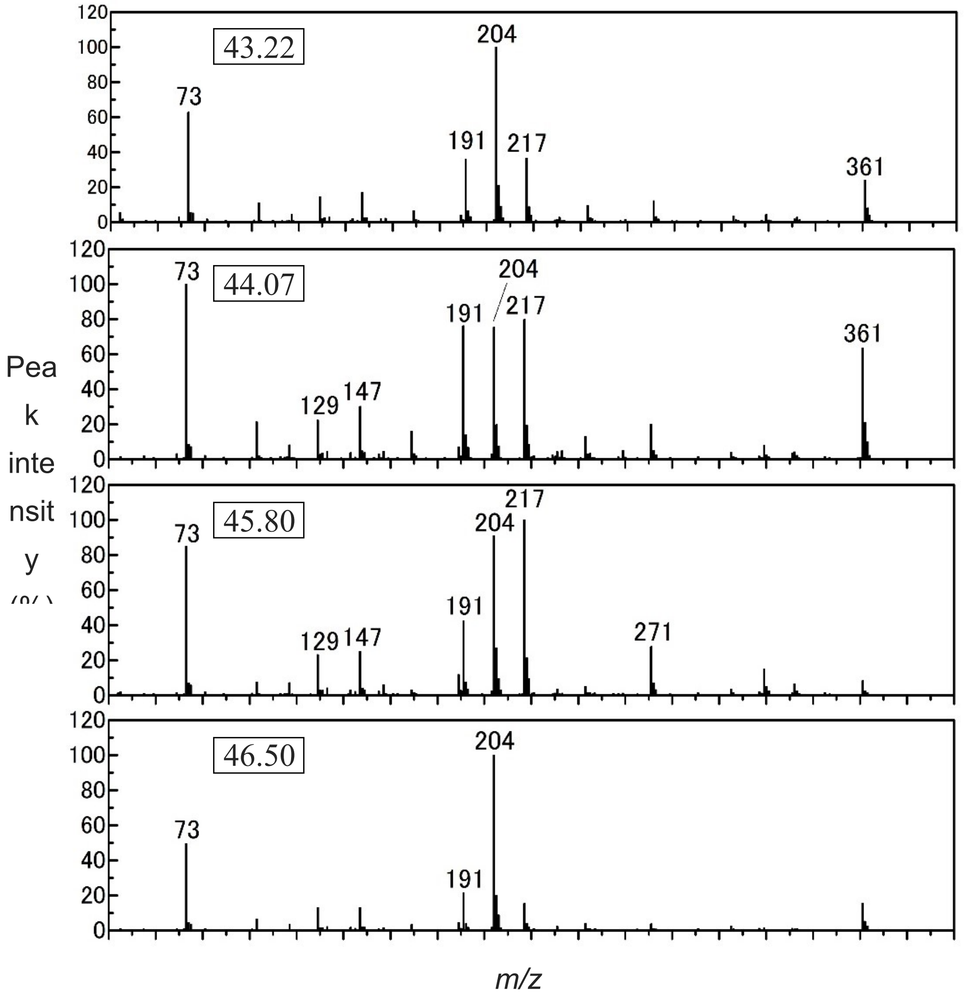

Fig. S5. The mass spectra of unidentified peaks observed in the GC-MS analysis of trimethylsilylated ChCl/glucose DES (1:1 molar ratio) after heating at 130°C for 180 min, with their retention times (min) in the upper left. All mass spectra are similar to those of the identified disaccharides (1)-(12) in Figs. S2 and S3, suggesting that the unidentified peaks correspond to similar disaccharides.

Table S1. Glucose residual rate (%) and the yield (wt%) of major glucose-derived products obtained upon heating ChCl/glucose DES (1:1 molar ratio) at 100−160 °C for 0−180 min. See experimental section for methods for calculating the yields.

Heating time (min)	100°C			130°C			160°C		
	Glucose residual rate (%)	Oligomer yield (%)	5-HMF yield (%)	Glucose residual rate (%)	Oligomer yield (%)	5-HMF yield (%)	Glucose residual rate (%)	Oligomer yield (%)	5-HMF yield (%)
0	100	0.5	ND	100	0.4	ND	100	0.5	ND
10	–	–	–	–	–	–	30.5	64.4	–
20	–	–	–	–	–	–	–	–	4.4
30	–	–	–	–	–	–	9.92	36.0	2.6
60	100	2.7	ND	76.9	46.7	0.2	6.19	28.2	1.9
120	98.7	4.8	ND	53.4	69.8	0.5	2.22	16.7	0.9
180	92.2	22.9	ND	32.2	70.3	0.5	2.08	3.7	0.6

ND: Not detected.

Table S2. Glucose residual rate (%) and the yield (wt%) of major glucose-derived products obtained upon heating neat glucose at 100−160 °C for 0−180 min. See experimental section for methods for calculating the yields.

Heating time (min)	100°C			130°C			160°C		
	Glucose residual rate	Oligomer	5-HMF	Glucose residual rate	Oligomer	5-HMF yield	Glucose residual rate	Oligomer	5-HMF
0	100	0.5	ND	100	0.4	ND	100	0.2	ND
10	–	–	–	–	–	–	66.2	66.9	–
20	–	–	–	–	–	–	–	77.5	0.047
30	–	–	–	–	–	–	25.2	–	–
40	–	–	–	–	–	–	–	–	0.057
60	96.8	0.4	ND	91.4	21.8	0.005	12.6	45.8	0.042
120	100.1	0.4	ND	74.1	51.7	0.009	5.2	25.2	0.034
180	95.2	0.4	ND	58.4	69.6	0.007	3.7	19.1	0.031

ND: Not detected.

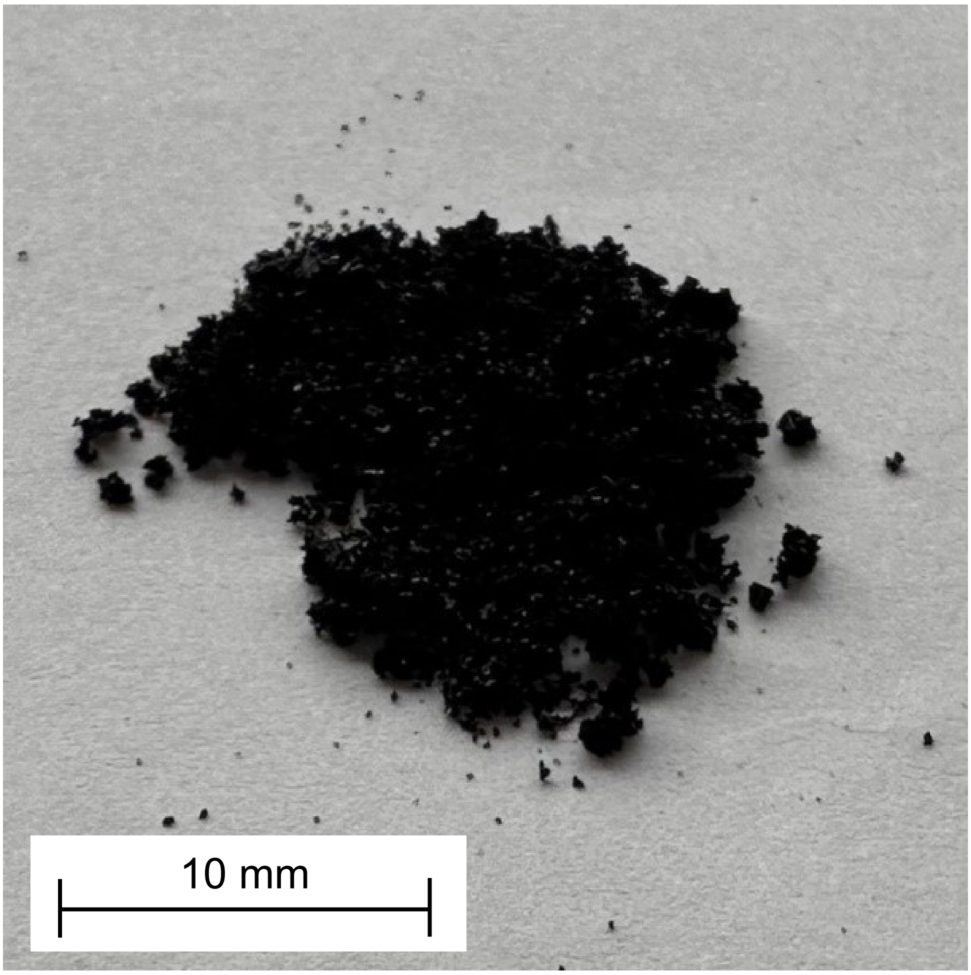

Fig. S6. The water-insoluble portion after heating of ChCl/glucose DES (1:1 molar ratio) at 160℃ for 180 min.
